# The combination of resveratrol and *Bletilla striata* polysaccharide decreases inflammatory markers of early osteoarthritis knee and the preliminary results on LPS‐induced OA rats

**DOI:** 10.1002/btm2.10431

**Published:** 2022-10-27

**Authors:** Tzu‐Chieh Lin, Jhih‐Ni Lin, I‐Hsuan Yang, Christina Soong, Ya‐Jyun Liang, Subhaini Jakfar, Chun‐Che Yen, Hwa‐Chang Liu, Hsuan‐Yu Chen, Feng‐Huei Lin

**Affiliations:** ^1^ Department of Biomedical Engineering, College of Medicine and College of Engineering National Taiwan University Taipei Taiwan; ^2^ Institute of Biomedical Engineering and Nanomedicine National Health Research Institutes Miaoli Taiwan; ^3^ Kartigen Biomedical Inc. Taipei Taiwan; ^4^ Department of Orthopaedic Surgery Taiwan Adventist Hospital Taipei Taiwan; ^5^ Department of Orthopaedic Surgery National Taiwan University College of Medicine and National Taiwan University Hospital Taipei Taiwan

**Keywords:** articular cartilage, *Bletilla striata*, chondrocytes, inflammation, osteoarthritis, oxidative stress, resveratrol

## Abstract

Osteoarthritis (OA) of the knee is characterized by progressive deterioration and loss of articular cartilage with associatedstructural and performance changes in the entire joint, and current treatments for OA only aim to relieve symptoms, rather than to prevent or reverse disease progression. Recently, treatments targeting “early osteoarthritis” (EOA) have attracted attention. However, during EOA stage, chondrocytes may change behaviors to express pro‐inflammatory cytokines and free radicals, which would cause detrimental effects to the synovial cavity and further cartilage wear. In this study, we combined resveratrol (Res) and *Bletilla striata* polysaccharide (BSP) as anti‐inflammatories and antioxidants to diffuse free radicals and to alleviate inflammation from the synovial cavity both short term and long term. The current study introduced a new method for harvesting BSP from as‐received *Bletilla striata* to achieve high yields, shortened extraction times, and maintained structure/functions. In addition, it combined Res and home‐extracted BSP (Res‐BSP) to alleviate oxidative stress and inflammation in a Lipopolysaccharide (LPS)‐induced OA model. The gene expressions of inflammatory genes iNOs, IL‐1β, IL‐6, and MMP‐13 were upregulated 5.7‐fold, 6.5‐fold, 8.6‐fold, and 4.5‐fold, respectively on OA‐like chondrocytes and the gene expressions were significantly downregulated to 3.3‐fold, 2.1‐fold, 4.9‐fold, and 0.1‐fold, respectively, once OA‐like chondrocytes were treated with Res‐BSP (*p* < 0.05, compared with OA‐like chondrocytes). The gene expressions of chondrogenic genes TGFβ1, SOX9, and type II collagen were downregulated by 0.8‐fold, 2.2‐fold, and 0.8‐fold, respectively, based on the control group as a baseline. While it was significantly upregulated by 3.4‐fold, 0.32‐fold, and 0.4‐fold, respectively, once OA‐like chondrocytes were treated with Res‐BSP. (*p* < 0.05, compared with OA‐like chondrocytes). Finally, we elucidated the role of Res‐BSP in EOA in suppressing COX‐2 and activating p‐Smad 2/3 and p‐Erk1/2. We believe that the combination of Res and BSP has great potential as an alternative therapeutic strategy for EOA treatment in future.

## 1 INTRODUCTION

Osteoarthritis (OA) of knee, known as degenerative synovial joint disease, is increasing in aging population and exerts substantial risk for pain, stiffness, fall and disability in elderly.^1^ The World Health Organization (WHO) Global Burden of Disease Study reported 26.6% increase in the costs of OA knee on healthcare systems from 1990 to 2010 in 21 epidemiological regions worldwide.^2^ Degenerative joint diseases will impact at least 130 million people globally by 2050 while world's population continues aging.^3^ Articular cartilage (AC), which covers the ends of bones forming synovial joint, facilitates load transmission across articular surfaces while permitting almost friction‐free movement and minimizing pressure on the underlying subchondral bone.^4^, ^5^ OA is characterized by progressive deterioration and loss of AC with associated structural and performance changes throughout the entire joint, including the synovium, periarticular ligaments, meniscus, and subchondral bone.^4^, ^6^ The deterioration of AC is slow during OA progression due to the opposite effects of wearing and healing on the cartilage.^7^, ^8^ Recently, many international scientific societies have demonstrated great interest in treatments targeting “early osteoarthritis” (EOA).^9^, ^10^ Chondrocytes may change behaviors to express pro‐inflammatory cytokines and free radicals, which would cause detrimental effects to the synovial cavity and further cartilage wear during EOA stage. It is important to treat patients with EOA to prevent disease progression and structural damages  associated with later stage of OA.^9^, ^10^

Resveratrol belongs to polyphenols' stilbenoids group, which possesses two phenol rings linked to each other by an ethylene bridge. This natural polyphenol has been found in more than 70 plants, which was reported very abundant in grapes' skin and seeds.^11^ It is a phytoalexin against pathogens, including bacteria and fungi.^12^ Resveratrol exhibits antioxidant and anti‐inflammatory properties.^11^, ^12^, ^13^, ^14^ It has been proven to be against IL‐1β‐induced catabolic effects and to prevent chondrocyte apoptosis by its inhibition of mitochondrial membrane depolarization and ATP depletion.^15^, ^16^ Although resveratrol exhibits good antioxidant and anti‐inflammatory activities compared with other common food additives,^17^, ^18^ and it is also available as a safe dietary supplement in contrast to other natural compounds.^19^ However, the bioavailability of resveratrol is lower by oral administration; and the half‐life of resveratrol is short by local delivery.^20^ Therefore, other compounds with long‐term anti‐inflammatory and antioxidant functions should be combined with resveratrol for therapy.

*Bletilla striata*, commonly known as the hyacinth orchid, is widely distributed in Southern and Eastern Asian countries and has been widely used as a traditional medicinal herb. Wang et al. found that a polysaccharide extracted from *Bletilla striata*, called *Bletilla striata* polysaccharide (BSP), is a di‐saccharide with repeated sequences of (1→2)‐α‐D‐mannopyranose and (1→4)‐β‐D‐glucopyranose residues, with a molar ratio of 2.4:1, molecular size of 135 kDa, and high water solubility. Many studies have reported that BSP has antioxidative, anti‐inflammatory, and anti‐aging properties.^21^, ^22^, ^23^ Studies by Lai et al. showed that BSP has chondro‐protective effects against H_2_O_2_‐induced oxidative stress by downregulating inflammatory markers such as IL‐1β and TNF‐α.^24^

In the study, BSP was extracted from *Bletilla striata* using a lab‐designed method. We hypothesized that the combination of resveratrol and BSP (Res‐BSP) with the functions of anti‐inflammation and antioxidant can diffuse free radicals to alleviate inflammation from the synovial cavity to achieve short‐term and long‐term effects, respectively, to resolve the deleterious environment of the synovial cavity during EOA.

## 2 MATERIALS AND METHODS

### 2.1 Experimental design

**FIGURE 1:**
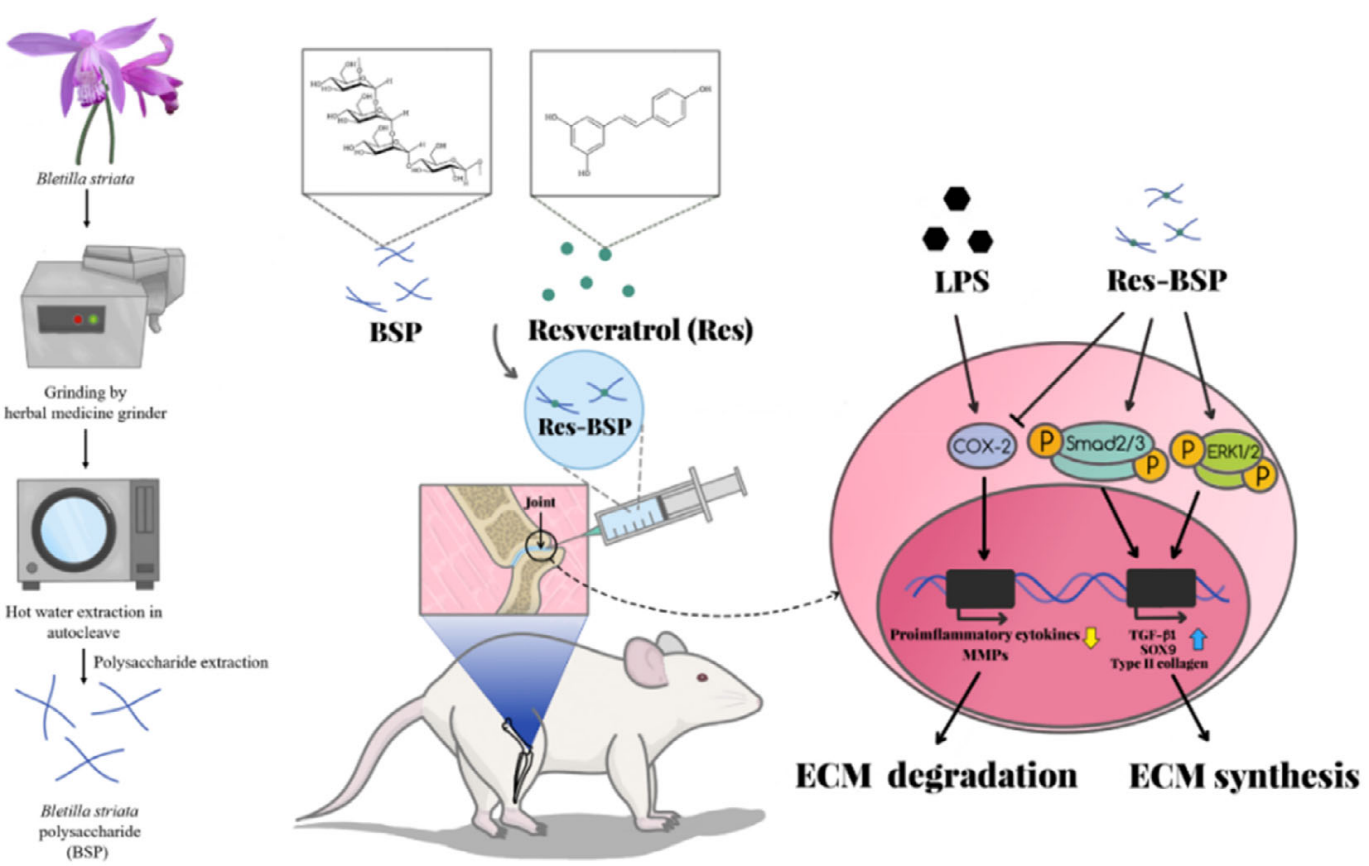
The scheme is the overall concept and experimental design. The BSP was extracted from as‐received *Bletilla Striata* by a lab‐designed method. The efficacy of Res‐BSP on LPS‐induced OA‐like chondrocyte is examined by RT‐qPCR, histo‐immuno‐chemical stain, and Western blot. The therapeutic efficiency of Res‐BSP on the early OA rat was evaluated by MRI and histology sectioning. BSP, *Bletilla striata* polysaccharide; LPS, lipopolysaccharide; MRI, magnetic resonance imaging; OA, osteoarthritis.

The overall experiment design is depicted in Figure 1. The BSP would be extracted and characterized by Fourier transform infrared spectroscopy (FTIR) and nuclear magnetic resonance (NMR) to identify functional groups and molecular structure, respectively. The cell viability and cell survival rate of Res‐BSP would be evaluated by WST‐1 and live/dead staining, respectively. Lipopolysaccharide (LPS) was used to induce chondrocytes into OA‐like chondrocytes; where the gene expression and ECM secretion would be checked by RT‐qPCR and histo‐immuno‐chemical stain, respectively, to confirm the OA‐like chondrocytes to be induced successfully. The induced pathway in the signal transduction would be further examined by Western blot. The SD rat intra‐articularly injected by LPS served as early OA model; and then delivered Res‐BSP by intra‐synovial injection once a week until sacrificed. The therapeutic efficiency of Res‐BSP on the early OA rat was evaluated by magnetic resonance imaging (MRI) and histology sectioning. The results would be analyzed by Osteoarthritis Research Society International (OARSI) scores to translate the results into a grade. The subchronic safety (8 weeks) of Res‐BSP on the experimental animal was evaluated by blood element and serological analysis.^25^ The independent variable is the treatment, such as resveratrol, BSP, Res‐BSP. The dependent variables are the response of treated chondrocyte and treated cartilage, such as the gene expression, ECM secretion, cartilage thickness change. The control group of in vitro study is normal cultured articular chondrocyte. The control group of in vivo study is normal knee joint of rat.

### 2.2 Extraction of polysaccharide from *Bletilla striata*

A novel method to isolate and purify BSP from as‐received *Bletilla striata* was utilized. Briefly, 100 g dry *Bletilla striata* purchased from a local herbal medicine pharmacy was chopped into pieces and ground into fine powder by a roll‐mill. The fine *Bletilla striata* powder was soaked in 1000 ml of double distilled water and placed in an autoclave for 30 min. The mixture was centrifuged at 6000 rpm for 5 min, and the supernatant was collected for later processing. 95% ethanol (Honeywell, 32205) was added into the previously collected supernatant at a ratio of 4:1 v/v. This solution was kept still overnight to precipitate the BSP from the supernatant. The resultant precipitate was collected with a 140‐mesh sieve and resuspended in double distilled water. Sevag reagent (chloroform and n‐butyl alcohol at a ratio of 4:1 [v/v]) was added to the resuspended precipitate at a volume ratio of 2:1 at room temperature and well‐mixed using a magnetic stirrer. It was kept still for 30 min to separate into two layers; proteins were bound to the Sevag reagent and sank down to the bottom layer, which was discarded. The top layer was collected and dialyzed (60035515, Orange Scientific) with a cutoff of 3500 Da. The dialyzed fraction was then lyophilized (FCU‐1200, Eyela) and stored at 4°C for later use.

### 2.3 Material characterization and analysis

The molecular structure of the extracted BSP was analyzed with ^13^C NMR and ^1^H NMR. The functional groups of the BSP were analyzed with a Fourier transform infrared spectrometer (JASCO 410) with a scanning range of 400 cm^−1^ to 4000 cm^−1^ at a scanning rate of 400 nm/min. The test sample was mixed with KBr in a 1:9 ratio and pressed into an aluminum disk with a pressure of 10 MPa.

### 2.4 Isolation and culture of porcine chondrocytes

Fresh porcine stifle joints were purchased from a traditional market and kept whole until chondrocytes were isolated under aseptic conditions. Full‐thickness porcine AC was minced and washed three times with sterilized PBS containing 5% antibiotics (Gibco, 15240‐062), followed by digestion with 0.5 U/ml collagenase (Serva, 17454) in high‐glucose Dulbecco's modified Eagle's medium (HG‐DMEM) (Sigma‐Aldrich, D5648) for 16 h at 37°C in a 5% CO_2_ incubator.

The resulting mixture of minced cartilage and collagenase was collected and centrifuged at 1300 rpm for 5 min. The supernatant was discarded and the precipitate was collected and gently washed by phosphate‐buffered saline (PBS) to remove the residual collagenase. The tissue debris was removed via filtration with a 100 μm strainer (352360, Corning), and chondrocytes in the filtrate were collected using centrifugation at 300*g* for 10 min. The isolated chondrocytes were cultured in HG‐DMEM supplemented with 10% fetal bovine serum (FBS; SH30084.03, Hyclone), 1.5 mg/ml sodium bicarbonate, 1% antibiotics, and 50 μg/ml L‐ascorbic acid (Sigma‐Aldrich, A5960) at 37°C in a 5% CO_2_ incubator. The medium was refreshed twice a week.

### 2.5 WST‐1 cell viability assay and live/dead staining

The effects of Res, BSP, and Res‐BSP on chondrocyte cell viability were evaluated using WST‐1 assays according to ISO 10993‐5 guideline. Zinc diethyldithiocarbamate (ZDEC; Sigma‐Aldrich, 329703) was used as the positive control and alumina (Al_2_O_3_; Sigma‐Aldrich, 11028) was used as the negative control^26^; these were referred to as control (+) and control (−), respectively. The chondrocytes were seeded in 96‐well culture plates (5 × 10^3^ cells/well) in DMEM containing 10% FBS at 37°C with 5% CO_2_ for 1 day. After the 1‐day culture, the medium was refreshed, and sample extraction solution was added to each well. After 24 h, the medium was removed and the 100 μl WST‐1 working solution was added per well. The plate was wrapped with aluminum foil to complete the reaction for 2 h in fully dark condition. The medium was then collected for further analysis with ELISA (Molecular Devices, SPECTRAmax Plus 384). The absorbance at 450 nm was measured and recorded. Each experiment was replicated six times. A live/dead staining kit was used to evaluate cell survival rate; this was conducted according to the manufacturer's instructions (Invitrogen, L3224).

### 2.6 OA‐like chondrocytes

Chondrocytes were seeded into six‐well culture plates at 2 × 10^5^ cells/well. After 18 h, the culture medium was changed to 2 ml of culture medium containing 10 μg/ml of LPS. After culturing for further 24 h, the chondrocytes are assumed to become OA‐like chondrocytes, which were collected for later use.

### 2.7 Evaluation of preventative efficacy of Res‐BSP treatment on OA‐like chondrocytes

OA‐like chondrocytes were seeded on a six‐well culture plate at a density of 2 × 10^5^ cells/well. After culturing for 1 day, the medium was changed to medium containing resveratrol (Res), *Bletilla striata* polysaccharides (BSP), or Res and BSP (Res‐BSP).

### 2.8 Gene expression of OA‐like chondrocytes

The target genes of the RT‐qPCR experiment were IL‐1β (Ss03393804_m1), IL‐6 (Ss03384604_u1), MMP13 (Ss03373279_m1), iNOS (Ss03374608_u1), TGF‐β1 (Ss03382325_u1), SOX9(Ss03392406_m1), and type II collagen (Ss03373342_g1). The reactions were performed with a Roche LightCycler® 96 System. Target gene expression was normalized to glyceraldehyde‐3‐phosphate dehydrogenase (GAPDH) (Ss03375435_u1). The relative mRNA expression of each target gene was determined using the −∆∆Ct method.

### 2.9 Alcian blue staining, immunocytochemistry staining, and Western blot analysis

Alcian blue reagent (1% Alcian blue 8GX [Sigma‐Aldrich, A5268] in 3% glacial acetic acid) was added to the chondrocytes and reacted for 90 min. The bound dye was eluted using a dissociation solution (33% n‐propanol [JT Baker, 9086‐1] with 4 M guanidine hydrochloride [Sigma‐Aldrich, G3272]). The OD value of the eluted solution was measured using an ELISA reader at the wavelength of 595 nm.^27^ Immunofluorescence images were analyzed using HCImage version 4.2.0 (Hamamatsu, ORCA ER). Fluorescence intensities of immunofluorescence images were quantified using ImageJ software. Membranes were incubated for 1 h at room temperature with antibodies specific for cyclooxygenase 2 (COX‐2) (ab15194, Abcam), p‐smad2/3 (phospho‐S467) (orb304593, biorbyt), and α‐tubulin (T5168, Sigma‐Aldrich). After three 10‐min washes, the blots were probed with horseradish peroxidase‐conjugated secondary antibody. Densitometric analysis of protein bands was performed using ChemiDoc XRS Plus image analysis software (Bio‐Rad Laboratories).

### 2.10 Animal model of early OA and MRI

Male Sprague–Dawley (SD) rats (6 weeks of age) were used. SD rats were purchased from BioLASCO. The animal study was approved by National Taiwan University, College of Medicine, Institutional Animal Care and Use Committee (IACUC number 20200031). SD rats were divided into four groups: control (Control), sham (Sham), LPS‐induced (LPS), and experimental (Res‐BSP). Each group contained six SD rats (*n* = 6). The LPS and Res‐BSP groups underwent intra‐articular injection with LPS solution (0.3 mg/50 μl saline per rat per week, for 8 weeks) to create an EOA model.^28^ Res‐BSP rats received Res‐BSP injections on the second day of LPS injection. The rats were examined with Bruker Biospec 7 T MRI (Bruker Corporation) before being euthanized. A total of 128 adjoining slices (thickness: 94 mm) were obtained with a 26 × 26 mm^2^ field of view and an image matrix of 512 × 512 pixels, generating a 51 × 51 × 94 mm^3^ voxel. A sagittal reconstruction of the data was used, as this plane allows better visualization of all knee cartilage compartments by minimizing partial volume effects.

All rats were euthanized with CO_2_ at the 8th week. Cardiac puncture was done with a 23G needle to harvest the blood for serological and blood element analysis. Safranin O/fast green staining was examined under optical microscope.

### 2.11 Histopathology and blood chemistry analysis

After rats were euthanized, the knee joints were obtained and fixed overnight in 10% neutral buffered formalin. They were then decalcified with 10% formic acid for 72 hours before being embedded in paraffin. About 6‐μm‐thick sagittal sections were made and stained with Safranin O/fast green stain. The results of the histological examination were evaluated using the OARSI scoring system.^29^ The safety of Res‐BSP in vivo was evaluated using blood element and serological analysis. Blood was collected using cardiac puncture after euthanization. Serum was obtained and centrifuged at 1300*g* at 4°C for 15 min and then stored at −80°C. Aspartate aminotransferase (AST), alanine aminotransferase (ALT), blood urea nitrogen (BUN), creatinine (CRE), and total protein (TP) were measured during biochemical testing.

### 2.12 Statistical analysis

Statistical data are expressed as mean ± standard deviation. Statistical analysis was performed using one‐way ANOVA with Tukey's multiple comparisons test; *p* < 0.05 (*) was considered statistically significant.

## 3 RESULTS

### 3.1 FTIR spectrometric analysis of BSP

In the study, we developed a new method to effectively isolate and purify BSP from as‐received *Bletilla striata*. The inclusion of autoclaving not only shortens extraction time, but also obtains BSP at high yields. Neither proteins nor nucleic acids were detected in the purified BSP.

**FIGURE 2:**
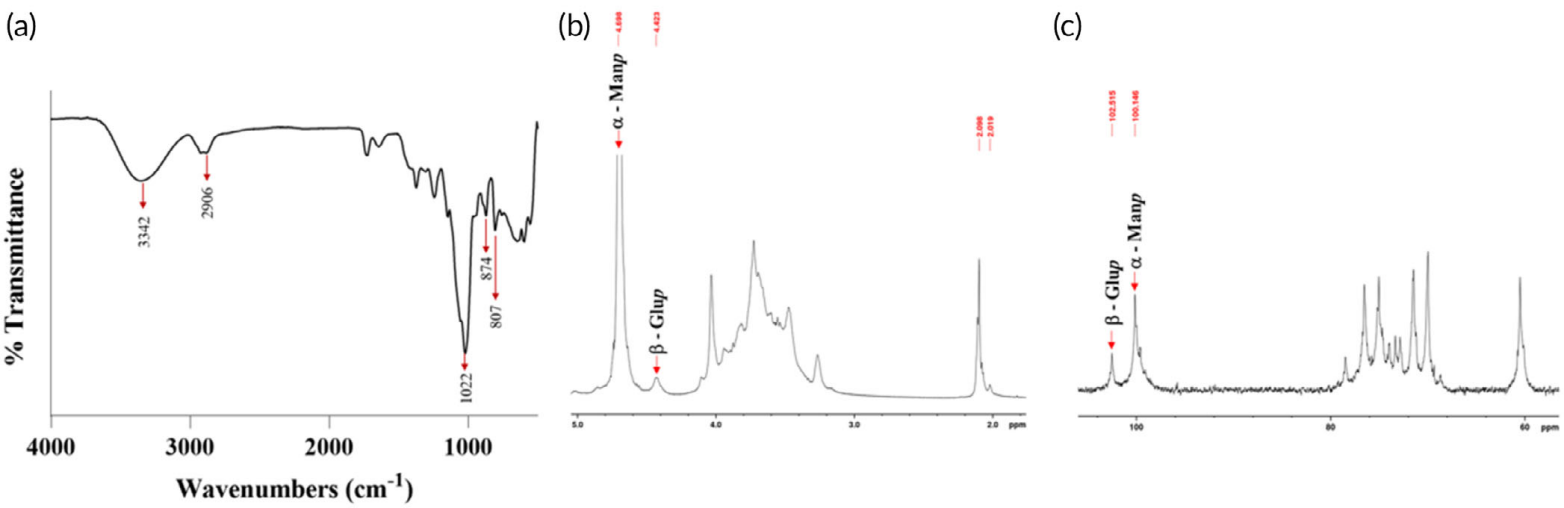
Characterization of BSP. (a) FTIR spectrum, (b) 1H NMR spectrum, and (c) 13C NMR spectrum. BSP, *Bletilla striata* polysaccharide; FTIR, Fourier transform infrared spectroscopy; NMR, nuclear magnetic resonance.

The FTIR pattern of BSP is shown in Figure 2a. The broad band at 3342 cm^−1^ was attributed to hydroxyl groups. The band at 2906 cm^−1^ likely corresponds to C−H stretching of the alkane. The absorption band at 1022 cm^−1^ indicated that the BSP was pyran‐glycosylated. The band at 874 cm^−1^ was attributed to β‐glucose residues and the band at 807 cm^−1^ was attributed to mannose residues.

### 3.2 NMR analysis of BSP

Figure 2b shows the ^1^H NMR spectrum of the extracted BSP, where the signal at 4.56 ppm was attributed to a β‐glucopyranosyl residue. The signals at 2.098 and 2.019 ppm belonged to a methyl and acetyl groups, respectively. Signals at 4.423 and 4.698 ppm were assigned to a β‐glucopyranosyl residue and α‐mannopyranosyl residue, respectively.

Figure 2c shows the ^13^C NMR spectrum of the extracted BSP. The anomeric signal at 100.146 ppm was attributed to an α‐mannopyranosyl residue. The anomeric signal at 102.515 ppm was assigned to a β‐glucopyranosyl residue.

### 3.3 WST‐1 test

**FIGURE 3:**
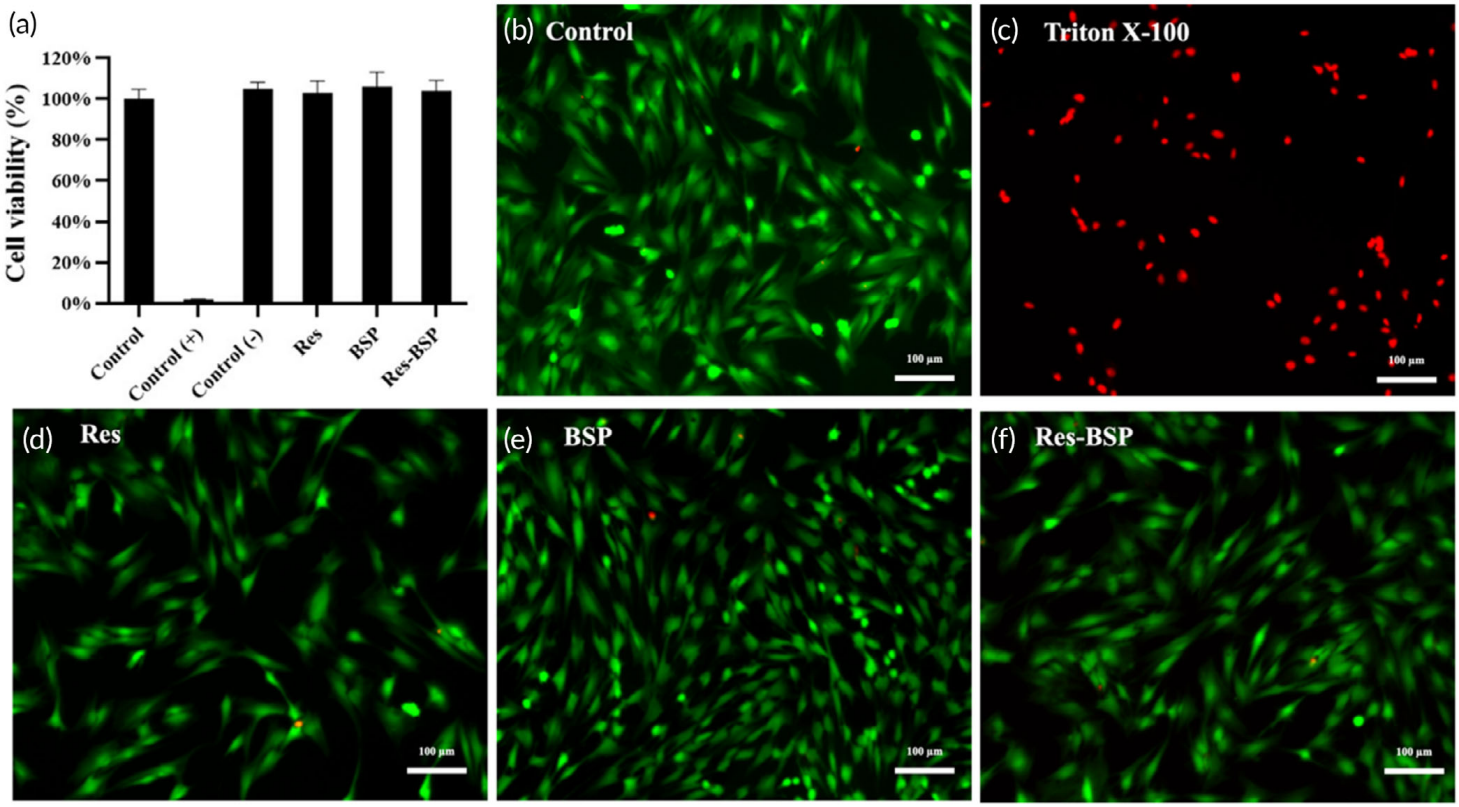
(a) The results of WST‐1 assays of chondrocytes treated with Res, BSP, and Res‐BSP; that is related to the cell number and would be in terms of cytotoxicity. *n* = 6. The results of live/dead staining of chondrocyte treated with (b) normal medium as the control group, (c) Triton X‐100, (d) Resveratrol, Res (e) BSP and (f) the combination of resveratrol and BSP, Res‐BSP; where the red color is dead cells and the green color is living cells. Scale bars = 100 μm; original magnification of 100×.

The effects of Res, BSP, and Res‐BSP on chondrocyte cell proliferation were evaluated by WST‐1 as shown in Figure 3a. The OD value showed no significant difference between the control, experimental, and negative control groups. However, the positive control had a much lower OD value, which was close to the baseline value. Based on ISO 10993‐5, the designed formula did not have cytotoxic potential.

### 3.4 Live/dead staining

Live/dead assays were used to evaluate the biocompatibility of resveratrol, BSP, and Res‐BSP. During live/dead staining, Calcein AM reacts with extracellular esterase and turns green to indicate living cells. EthD‐1 binds to the DNA of dead cells to emit red fluorescence. Figure 3b–f showed that there were no significant differences in the images from live/dead staining of the control, Res, BSP, and Res‐BSP groups. The results further confirmed that the designed formulae were not toxic to the chondrocytes.

### 3.5 Gene expression evaluation with RT‐qPCR

iNOS, IL‐1β, IL‐6, and MMP13 expression levels were analyzed to assess effects on inflammation‐related genes. TGF‐β1, SOX9, and type II collagen expression levels were analyzed to assess effects on chondrogenesis‐related genes. The ∆∆Ct of the control group was used as a baseline to normalize the other groups.

**FIGURE 4:**
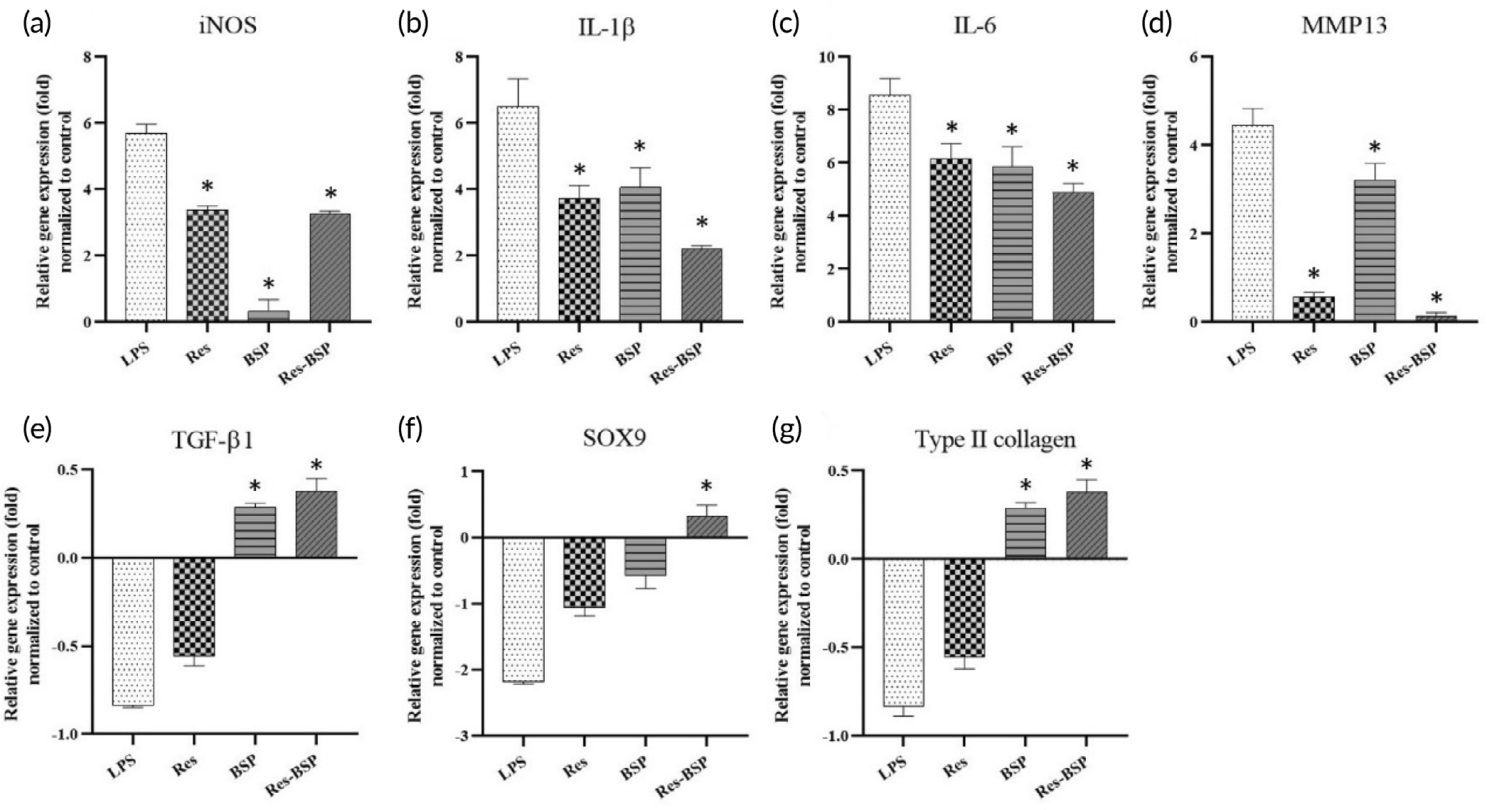
Gene expression of chondrocyte; those were normalized to housekeeping gene (GAPDH) and control as ΔΔCt. (a) iNOS (b) IL‐1β, (c) IL‐6, (d) MMP13, (e) TGF‐β1, (f) SOX9, and (g) type II collagen. *n* = 6, *p < 0.05 compared with LPS group.

The chondrocytes were induced to OA‐like chondrocytes with LPS. As shown in Figure 4a, iNOS gene was significantly upregulated 5.7‐fold in OA‐like chondrocytes. It was downregulated to 3.4‐fold, 0.3‐fold, and 3.3‐fold once OA‐like chondrocytes were treated with Res, BSP, or Res‐BSP, respectively (*p* < 0.01). IL‐1β was significantly upregulated by 6.5‐fold in OA‐like chondrocytes. It was downregulated to 3.7‐fold, 4.1‐fold, and 2.1‐fold in the Res, BSP, and Res‐BSP groups, respectively, as shown in Figure 4b. Figure 4c shows the gene expression of IL‐6 which showed similar results as the previous inflammation‐related genes. IL‐6 expression in OA‐like chondrocytes was upregulated 8.6‐fold; whilst it was downregulated to 6.2‐fold, 5.8‐fold, and 4.9‐fold once OA‐like chondrocytes were treated with Res, BSP, and Res‐BSP, respectively. Figure 4d shows the gene expression of MMP13. The results showed that MMP13 was upregulated in OA‐like chondrocytes by 4.5‐fold. After OA‐like chondrocytes were treated with Res, BSP, or Res‐BSP, MMP13 expression was downregulated to 0.6‐fold, 3.2‐fold, and 0.1‐fold, respectively.

With regards to chondrogenesis‐related genes, TGF‐β1 expression in OA‐like chondrocytes was downregulated by 0.8‐fold, based on the control group as a baseline. After OA‐like chondrocytes were treated with Res, the gene expression of TGF‐β1 was downregulated to 0.6‐fold, while it was upregulated by 0.3‐fold and 3.4‐fold in the BSP and Res‐BSP groups, respectively (Figure 4e). Figure 4f shows the expression SOX‐9, which was downregulated by 2.2‐fold, 1.1‐fold, and 0.6‐fold in the LPS, Res, and BSP groups, respectively. It was upregulated by 0.32‐fold after OA‐like chondrocytes were treated with Res‐BSP. The gene expression of type II collagen was downregulated by 0.8‐fold and 0.6‐fold in the LPS and Res groups, respectively. It was upregulated by 0.3‐fold and 0.4‐fold in the BSP and Res‐BSP groups, respectively (Figure 4g).

### 3.6 Alcian blue staining

**FIGURE 5:**
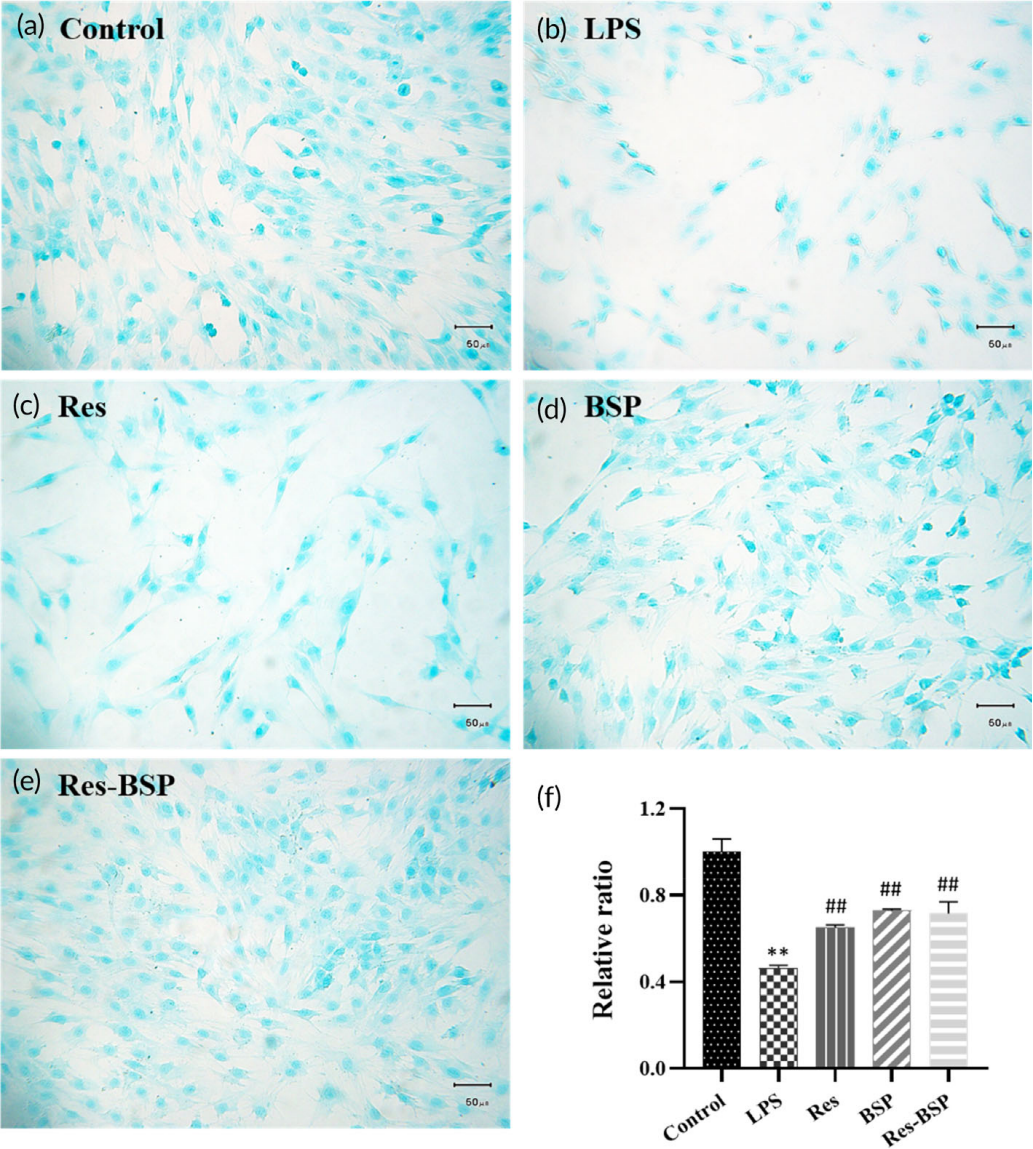
The results of Alcian blue staining; where (a) Control, (b) LPS, (c) Res, (d) BSP, (e) Res‐BSP, and (f) quantitative result of the Alcian blue staining by ImageJ software. *n* = 6, **p < 0.01 compared with control group; ##*p* < 0.01 compared with LPS group. Bars = 50 μm; original magnification of 200×. BSP, *Bletilla striata* polysaccharide; LPS, lipopolysaccharide.

Alcian blue stain binds to sulfated GAGs in the extracellular matrix (ECM) to turn blue. The control group highly secreted sulfated GAGs and had a deep blue color as shown in Figure 5a. The OA‐like chondrocytes showed a relatively light blue color as shown in Figure 5b. After treatment with Res, BSP, or Res‐BSP, OA‐like chondrocytes recovered to secrete more sulfated GAGs and became deeper blue as shown in Figure 5c, Figure 5d, and Figure 5e, respectively. The quantitative data generated by GraphPad Prism is summarized in Figure 5f.

### 3.7 Immunocytochemistry staining of type II collagen

**FIGURE 6:**
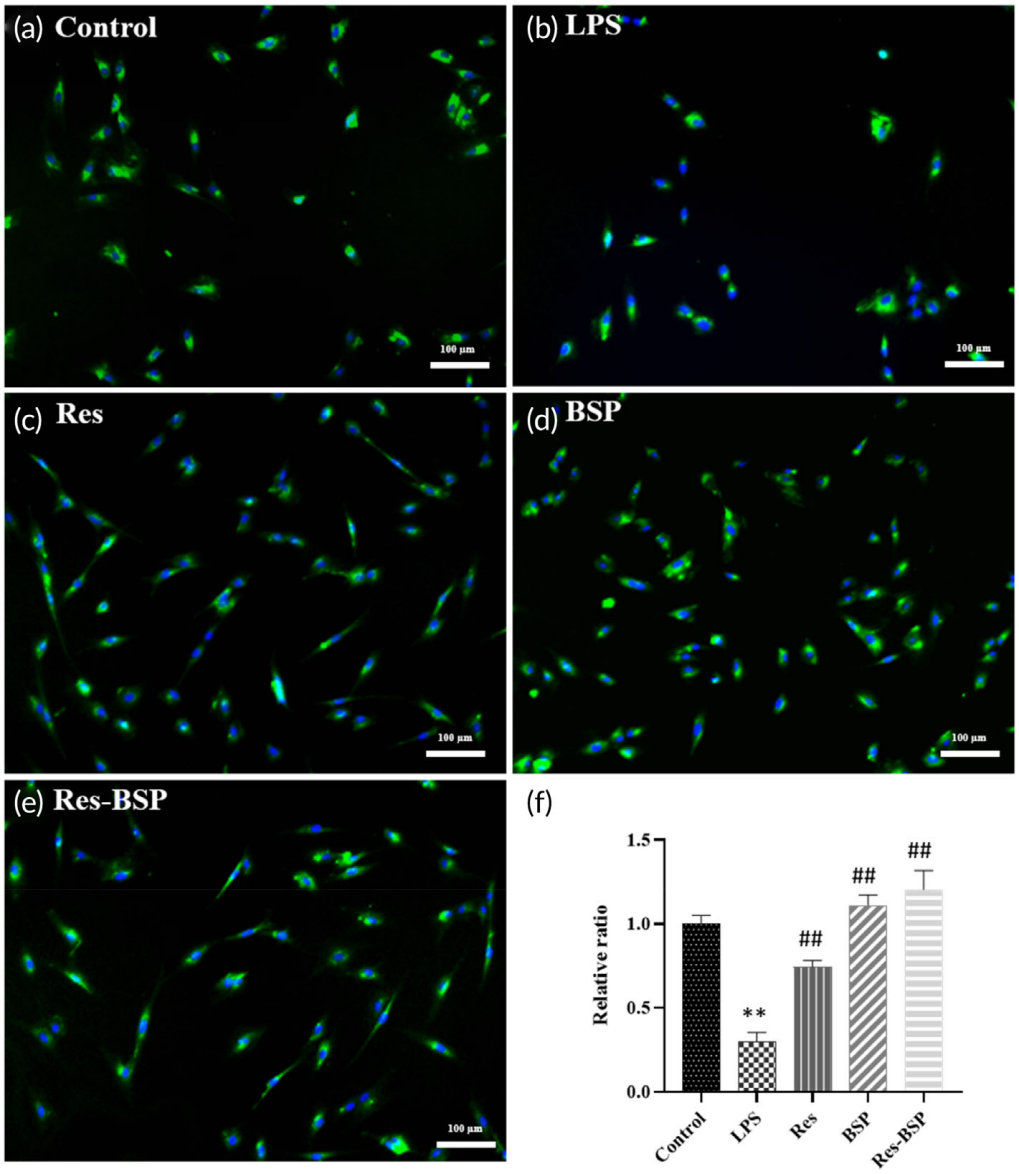
Immunocytochemistry staining of collagen type II on the chondrocyte, where the green color is the collagen type II and the blue color is the contrast stain of the nucleus. (a) Control, (b) LPS, (c) Res, (d) BSP, (e) Res‐BSP, and (f) the quantitative analysis of the results by ImageJ software. *n* = 6, **p < 0.01 compared with control group; ##*p* < 0.01 compared with LPS group. Bars = 50 μm; original magnification of 200×. BSP, *Bletilla striata* polysaccharide; LPS, lipopolysaccharide.

Figure 6 shows the results of immunocytochemistry staining of type II collagen, in which the green color indicates type II collagen (Figure 6a) and the blue color was a contrast stain for nuclei. OA‐like chondrocytes showed relatively weak secretion of type II collagen (Figure 6b), and secretion increased after treatment with Res, BSP, or Res‐BSP as shown in Figure 6c, Figure 6d, and Figure 6e, respectively. Figure 6f shows the quantitative summary of type II collagen secretion generated by GraphPad Prism.

### 3.8 Western blot

**FIGURE 7:**
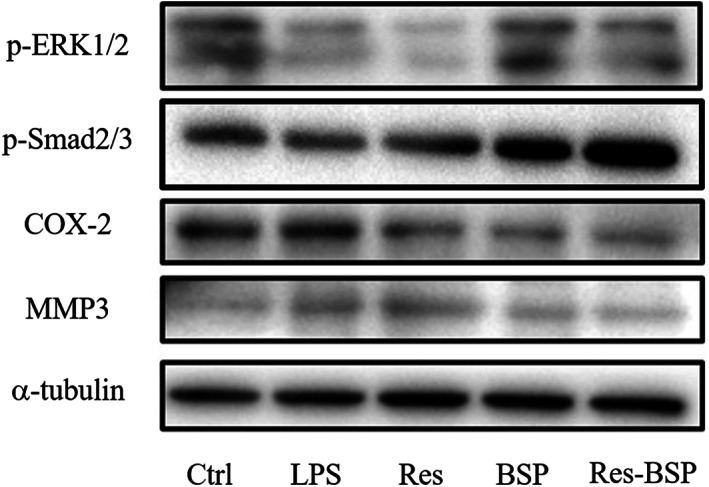
Western blot analysis of OA‐like chondrocytes induced by LPS and then treated by Res, BSP, and Res‐BSP. BSP, *Bletilla striata* polysaccharide; LPS, lipopolysaccharide; OA, osteoarthritis.

The top two rows of Figure 7 show the results of ERK1/2 and Smad2/3 expression; these were upregulated after OA‐like chondrocytes were treated with BSP or Res‐BSP. The rows next to ERK1/2 and Smad2/3 in Figure 7 show the expressions of COX‐2 and MMP3; these were upregulated after chondrocytes were treated with LPS. After treatment with Res, BSP, or Res‐BSP, the OA‐like chondrocytes showed downregulated expression of COX‐2 and MMP3. The bottom row shows α‐tubulin expression which was used as an internal control. The results of in vitro experiments demonstrated the combination of resveratrol and BSP to diffuse oxidative stress with short‐term and long‐term effects to alleviate the inflammation and ECM degradation via suppressing the COX‐2 and activating p‐Smad 2/3 and p‐Erk1/2 signal pathway in LPS‐induced OA model.

### 3.9 Magnetic resonance imaging

After 2 months, the knee of the rat was examined using MRI. A total of 128 adjoining slices with thicknesses of 94 mm were obtained in a field view of 26 × 26 mm^2^ by an image matrix of 512 × 512 pixels, generating a 51 × 51 × 94 mm^3^ voxel. A sagittal reconstruction of the data was chosen to minimize partial volume effects. The acquired volume completely covered the joint. The images were analyzed and translated by ImageJ software.

**FIGURE 8:**
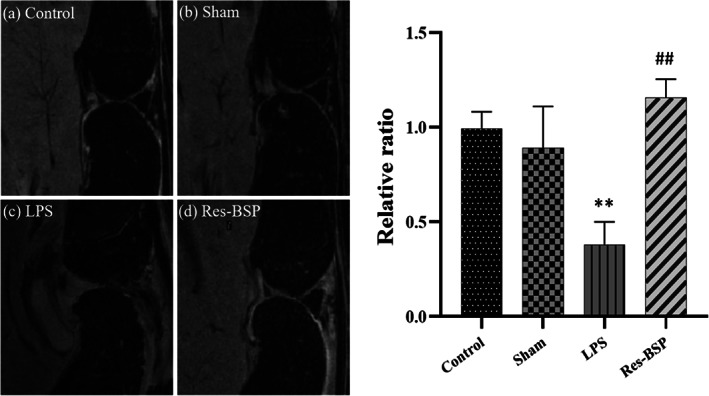
T2‐weighted MRI image of articular cartilage. (a) Control, (b) Sham, (c) LPS, (d) Res‐BSP, and (e) the quantitative results for all the groups interpreted by ImageJ; where the lower ratio indicated the higher damage of the articular cartilage. *n* = 6, **p < 0.01 compared with control group; ##*p* < 0.01 compared with LPS group. BSP, *Bletilla striata* polysaccharide; LPS, lipopolysaccharide; MRI, magnetic resonance imaging.

Figure 8a shows the control group. Figure 8b shows the sham group, where the knee of the rat was only treated with normal saline. Figure 8c shows the results of the LPS group, where rats received LPS injected into the knee to induce OA‐like morphology. Figure 8d shows the results of rats injected with LPS followed by Res‐BSP treatment. Figure 8e shows the quantitative summary for all groups generated by ImageJ, where the bright‐light image of the control group was indexed as 1, and the other groups were normalized to the control group to obtain a ratio. Lower ratios indicated higher degree of damage of the AC. The Res‐BSP group had the highest ratio from MRI analysis.

### 3.10 Histological analysis

**FIGURE 9:**
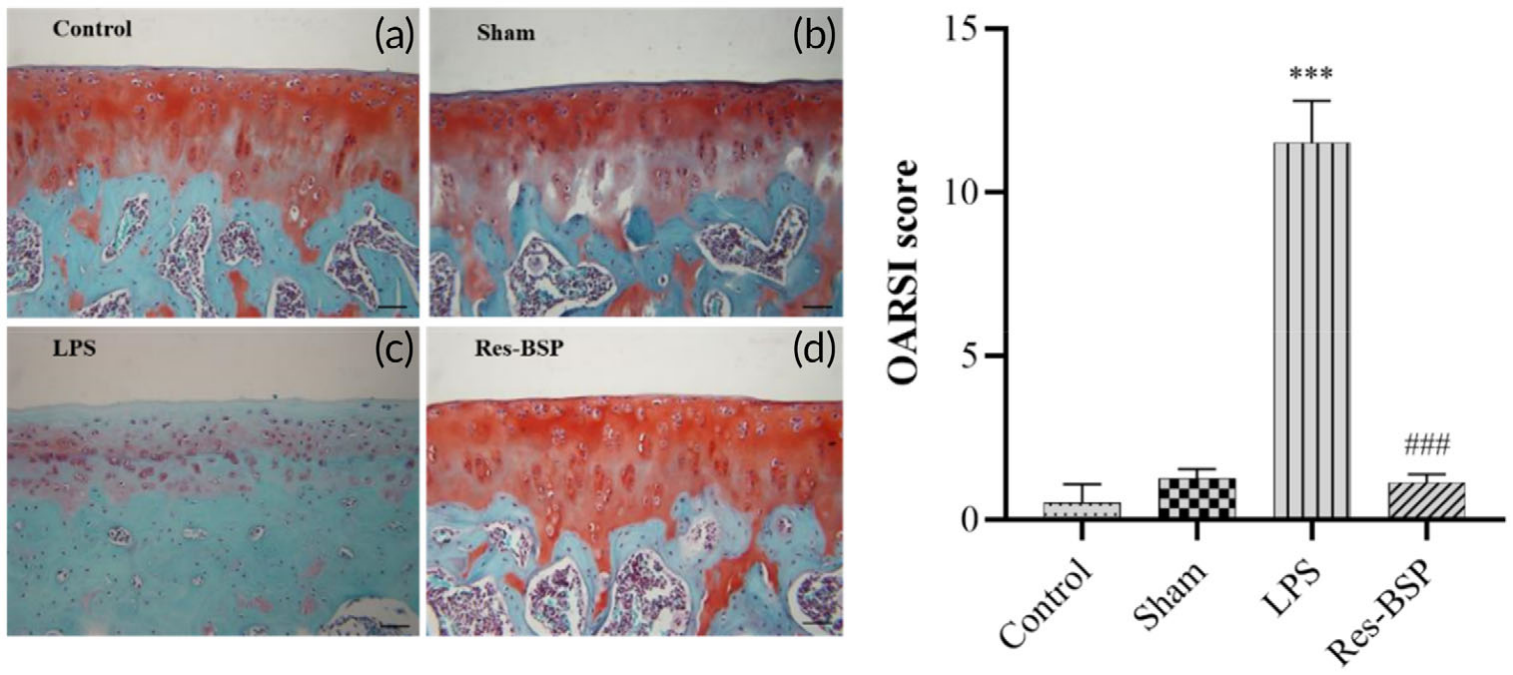
Safranin O staining of acidic proteoglycan on articular cartilage. (a) Control, (b) Sham, (c) LPS, (d) Res‐BSP, (e) Degree of joint damage was evaluated using the Osteoarthritis Research Society International (OARSI) scores. *n* = 6, ***p < 0.001, compared with control group. ###*p* < 0.001, compared with LPS group. BSP, *Bletilla striata* polysaccharide; LPS, lipopolysaccharide.

Figure 9 shows the results of histological sectioning with HE staining and Safranin‐O staining, followed by examination under an optical microscope. Figure 9a shows the control group, Figure 9b shows the sham group, Figure 9c shows the knee of an LPS‐induced OA rat, and Figure 9d shows an LPS‐induced OA rat that was further treated with Res‐BSP. The images were analyzed by ImageJ software and translated into OARSI scores. The scores were used to evaluate OA progression. Higher scores represented stronger OA progression. From the results shown in Figure 9e, the scores of the Res‐BSP group were similar to those of the control and sham groups.

Based on the results of MRI and histological analysis, we believe that Res‐BSP has the potential for protecting against and treating LPS‐induced OA. Based on the results of in vivo experiments, we believe that Res‐BSP has the potential for protecting against and treating LPS‐induced OA.

### 3.11 Blood chemistry analysis

The results of blood element and serological analyses are shown in Data S1. Values out of the normal range obtained from the control group are shown in red to indicate abnormality. In the LPS group, the ALT was abnormally high, likely due to liver damage from LPS. The values from blood element and serological analyses of the Res‐BSP group were all within normal ranges.

## 4 DISCUSSION

The initial onset of OA is due to imbalanced catabolism and anabolism in the ECM of cartilage. One hypothesis is that reactive oxygen species (ROS) in the synovial cavity may induce secretion of pro‐inflammatory cytokines to trigger the production of matrix metalloproteinases, which then causes cartilage ECM fragmentation and degradation that may result in EOA.^9^, ^30^ During EOA, chondrocytes may begin to only secrete short‐chain collagen fibers and HA molecules with low‐molecular weight, which do not have the mechanical properties for weight bearing.^31^ Under these circumstances, the chondrocytes may start expressing pro‐inflammatory cytokines and free radicals to create a deleterious environment that induces ECM degradation and leads to wearing of cartilage.^26^, ^32^ If we could introduce anti‐inflammatory and antioxidant polysaccharides or reagents during EOA to slow down OA progression and to create a healthy environment for the behavior‐changed chondrocytes, these may induce OA‐like chondrocytes to begin secreting qualified ECM to for cartilage support again, instead of progressing toward senescence or apoptosis.^28^ Generally, the degradation of polysaccharides is slow; this method alone may not be sufficient for creating a healthy environment for the OA‐like chondrocytes during the short term. Introducing an additional anti‐inflammatory and antioxidant reagent which can quickly diffuse free radicals and reduce the intensity of inflammation from the nonfriendly environment is necessary.^33^ In this study, a reagent and a polysaccharide which both functions as anti‐inflammatory and antioxidant agents were combined to diffuse free radicals and/or alleviate inflammation from the synovial cavity in the short term and long term, respectively.

Res was combined with BSP using local delivery to retain bioactivity and to remove ROS from the synovial cavity during EOA to prevent further ECM loss and to stop OA progression during the early phase. The chondrocytes were transformed to OA‐like chondrocytes using LPS. As shown in Figure 4a, the iNOS gene was significantly upregulated 5.7‐fold in OA‐like chondrocytes, and downregulated almost to baseline once OA‐like chondrocytes were treated with Res. The IL‐1β gene was significantly upregulated 6.5‐fold on OA‐like chondrocytes. It was downregulated to 3.7‐fold in the Res group, as shown in Figure 4b. Figure 4c shows the gene expression of IL‐6 which had similar results as the previous inflammatory‐related genes. Figure 4d shows the gene expression of MMP13. The results showed that MMP13 expression in OA‐like chondrocytes was upregulated 4.5‐fold. After OA‐like chondrocytes were treated with Res, MMP13 expression was downregulated to 0.6‐fold. With regards to chondrogenesis‐related genes, the expression of TGF‐β1, SOX9, and type II collagen in OA‐like chondrocytes were downregulated, and then upregulated after treatment with Res. Type II collagen and proteoglycan are the major components of the ECM of articular chondrocytes. As shown in Figures 5f and 6f, the ECM deposition of type II collagen and proteoglycan in LPS‐induced OA‐like chondrocytes decreased to 36% and 30%, respectively, compared to normal chondrocytes. They were upregulated to 65% and 74%, respectively, once OA‐like chondrocytes were treated with Res.

*Bletilla striata* polysaccharides (BSPs) are reported to remain in the synovial cavity for a long period of time due to the lack of specific enzyme which degrades polysaccharides.^21^, ^34^ In this study, we used BPS to serve as the long‐term polysaccharide to remove ROS from the synovial cavity during EOA. As shown in Figure 4a–d, the expression of inflammation‐related genes iNOS, IL‐1β, IL‐6, and MMP13 were downregulated to 0.3‐fold, 4‐fold, 5.8‐fold, and 3.2‐fold, respectively, after OA‐like chondrocytes were treated with BSP. As shown in Figure 4e–g, expression of chondrogenic‐related genes TGF‐β1, SOX9, and type II collagen were also upregulated when OA‐like chondrocytes were treated with BSP. As shown in Figures 5f and 6f, the ECM deposition of type II collagen and proteoglycan was upregulated to 72% and 111%, respectively, once OA‐like chondrocytes were treated with BSP.

Res‐BSP showed synergistic effects in anti‐inflammation and regenerative effects in OA‐like chondrocytes. As shown in Figure 4, the expression of inflammation‐related genes iNOS, IL‐1β, IL‐6, and MMP13 were downregulated to 3.2‐fold, 2.1‐fold, 4.9‐fold, and 0.1‐fold, respectively, after Res‐BSP treatment. The expression of chondrogenic‐related genes TGF‐β1, SOX9, and type II collagen were significantly upregulated when OA‐like chondrocytes were treated with Res‐BSP. As shown in Figures 5f and 6f, the ECM deposition of type II collagen and proteoglycan were upregulated to 72% and 120%, respectively, once OA‐like chondrocytes were treated with Res‐BSP.

The TGF‐β1 signaling cascade has been reported as an anabolic pathway in articular chondrocytes via the phosphorylation of the ERK1/2 and Smad2/3 cascades to promote chondrocyte proliferation and ECM synthesis.^35^, ^36^, ^37^ The expression levels of phoshpo‐ERK1/2(p‐ERK1/2) and phoshpo‐Smad2/3 (p‐Smad2/3) were significantly upregulated by BSP as well as by Res‐BSP. As shown in Figure 9, the expressions of p‐ERK1/2 and p‐Smad2/3 in OA‐like chondrocytes were downregulated and then upregulated after treatment with BSP or Res‐BSP. Several inflammatory cytokines are involved in pathophysiological processes associated with OA, such as interleukin (IL)‐6, IL‐1β, and tumor necrosis factor‐α (TNF‐α).^38^ The COX‐2/prostaglandin E2 (PGE2) pathway is a common pathway for inducing inflammation and ECM degradation. The expression levels of COX‐2 and MMP3 were upregulated by LPS compared to the control group and suppressed after BSP or Res‐BSP treatment. Although intra‐articular injection of hyaluronic acid has been widely used in clinical practice, its efficacy is still limited to protect AC from degeneration temporarily.^39^ The in vivo experimental results showed that Res‐BSP had anti‐inflammatory and chondrogenic‐promoting effects, which were further confirmed by MRI imaging (Figure 8) and Safranin‐O staining (Figure 9).

This study suggested that Res‐BSP could reverse LPS‐induced inflammation by upregulating chondrogenic genes (SOX9, type II collagen, and TGF‐β1) and downregulating inflammation‐related genes (iNOS, IL‐1β, IL‐6, and MMP13) via activation of TGF‐β1 and its downstream mediators Smad2/3 and ERK1/2. This can promote ECM synthesis and inhibit COX‐2‐mediated inflammation and ECM degradation.

In addition, we developed a novel method for isolating and purifying BSP from as‐received *Bletilla striata*. During the preparation process, *Bletilla striata* powder was ground into fine particles and BSP was extracted by autoclave to shorten extraction times. During BSP precipitation, the volume ratio of extracted BSP to 95% (v/v) ethanol was 4:1 rather than 3:1 as reported with other methods. The extracted BSP showed similar FTIR (Figure 2a), ^1^H spectrum (Figure 2b), and ^13^C NMR spectrum (Figure 2c) patterns to standard BSP. These results indicate that this novel method generates high BSP yield with short extraction times and does not affect BSP structure/functions.^21^, ^24^

The limitation of the study is that synovial cavity of rat is too small to observe the therapeutic progression of OA, and porcine or dogs would be better options for animal models in further study. MRI, histological analysis, and blood chemistry analysis have been done to strengthen the evidence of therapeutic effect. LPS resulted in marked production of polyclonal antibodies and induced the secretion of various mediators including IL‐12 and IFN‐γ involved in cellular immune responses, keeping the inflammatory pathways elevated in our animal model system to mimic OA, and OA of knee, which is the weight‐bearing joint, contained biomechanical factors. However, amplification of catabolic activity to increase the expression of inflammatory mediator is the major pathophysiological mechanism in EOA, and LPS animal model could help us create the pathological process of imaging and histology.

## 5 CONCLUSIONS

In conclusion, the current study demonstrated, firstly, a novel method for harvesting BSP from as‐received *Bletilla striata* with high yields, short extraction times, and no change in structures/functions. In addition, it showed that combined Res and BSP can diffuse oxidative stress both short term and long term to alleviate inflammation in an LPS‐induced OA model. Finally, this study elucidated that Res‐BSP exerts effects in EOA via suppression of COX‐2 and activation of p‐Smad2/3 and p‐ERK1/2. We believe that the combination of Res and BSP may be an alternative therapeutic strategy for EOA treatment.

## AUTHOR CONTRIBUTIONS

**Tzu‐Chieh Lin:** Data curation (equal); formal analysis (equal); investigation (equal); resources (equal). **Jhih‐Ni Lin:** Methodology (equal). **I‐Hsuan Yang:** Data curation (equal); formal analysis (equal). **Christina Soong:** Conceptualization (equal); writing – review and editing (equal). **Ya‐Jyun Liang:** Methodology (equal). **Subhaini Jakfar:** Data curation (equal); validation (equal); writing – review and editing (equal). **Chun‐Che Yen:** Project administration (equal); writing – original draft (equal). **Hwa‐Chang Liu:** Conceptualization (equal); supervision (equal). **Feng‐Huei Lin:** Conceptualization (equal); supervision (equal); writing – review and editing (equal). **Hsuan‐Yu Chen:** Conceptualization (equal); data curation (equal); funding acquisition (equal); validation (equal); writing – original draft (equal).

### PEER REVIEW

The peer review history for this article is available at https://publons.com/publon/10.1002/btm2.10431.

## Supporting information


**DATA S1** The result of hematological and serological analyses. *n* = 6, *p < 0.05 compared with control group

## Data Availability

The data that support the findings of this study are available on request from the corresponding author. The data are not publicly available due to privacy or ethical restrictions.
