# Supplementary material for: The combination of resveratrol and Bletilla striata polysaccharide decreases inflammatory markers of early osteoarthritis knee and the preliminary results on LPS‐induced OA rats
Source: Bioeng Transl Med. 2022 Oct 27;8(4):e10431. doi: 10.1002/btm2.10431 (PMC10354757; doi:10.1002/btm2.10431)
Supplement: Supplementary file 1 — DATA S1 The result of hematological and serological analyses. n = 6, *p < 0.05 compared with control group [file BTM2-8-e10431-s001.docx]

Supplement data 1 The result of hematological and serological analyses. n = 6, * p < 0.05 compared with control group.

|  | Control | Sham | LPS | Res-BSP |
| --- | --- | --- | --- | --- |
| RBC (M/ μL) | 9.38 ± 0.34 | 9.50 ± 0.45 | 9.92 ± 0.36 | 9.55 ± 0.37 |
| HGB (g/ dL) | 15.9 ± 1.92 | 16.6 ± 2.11 | 16.5 ± 1.78 | 16.9 ± 1.33 |
| HCT (%) | 47.9 ± 4.23 | 49.3 ± 3.56 | 48.3 ± 2.23 | 48.2 ± 4.01 |
| MCV (fL) | 51.1 ± 3.52 | 51.9 ± 2.88 | 48.7 ± 5.29 | 50.5 ± 3.21 |
| MCH (pg) | 17.0 ± 0.76 | 17.5 ± 0.68 | 16.6 ± 1.01 | 17.7 ± 0.35 |
| MCHC (g/ dL) | 33.2 ± 1.98 | 33.7 ± 1.11 | 34.2 ± 2.13 | 35.1 ± 2.07 |
| PLT (K/ μL) | 850 ± 84.80 | 898 ± 77.53 | 656 ± 87.09 | 804 ± 91.68 |
| WBC (K/ μL) | 4.57 ± 1.91 | 5.71 ± 1.58 | 4.78 ± 2.14 | 4.28 ± 1.06 |
| NEUT (K/ μL) | 0.91 ± 0.17 | 0.82 ± 0.24 | 0.65 ± 0.11 | 0.75 ± 0.19 |
| LYMPH (K/ μL) | 3.18 ± 1.25 | 4.56 ± 1.18 | 3.60 ± 2.01 | 3.07 ± 1.65 |
| MONO (K/ μL) | 0.30 ± 0.04 | 0.16 ± 0.03 | 0.12 ± 0.09 | 0.16 ± 0.08 |
| EO (K/ μL) | 0.15 ± 0.10 | 0.16 ± 0.07 | 0.41 ± 0.06 | 0.29 ± 0.10 |
| BASO (K/ μL) | 0.03 ± 0.01 | 0.01 ± 0.01 | 0.02 ± 0.01 | 0.01 ± 0.01 |
| AST (U/ L) | 130 ± 14.06 | 188 ± 15.37 | 198 ± 20.15 | 156 ± 17.55 |
| ALT (U/ L) | 57 ± 19.31 | 56 ± 12.66 | 121 ± 20.51 ^*^ | 54 ± 13.92 |
| CRE (mg/ dL) | 0.4 ± 0.10 | 0.4 ± 0.12 | 0.5 ± 0.11 | 0.5 ± 0.15 |
| BUN (mg/ dL) | 17 ± 4.16 | 19 ± 4.08 | 19 ± 5.33 | 16 ± 3.78 |
| RBC: red blood cell; HGB: hemoglobin; HCT: hematocrit; MCV: mean corpuscular volume; MCH: mean corpuscular hemoglobin; MCHC: mean corpuscular hemoglobin concentration; PLT: platelet; WBC: white blood cell; NEUT: neutrophil; LYMPH: lymphocyte; MONO: monocyte; EO: eosinophil; BASO: basophil; AST: Aspartate Transaminase; ALT: alanine aminotransferase; CRE: Creatinine; BUN: Blood Urea Nitrogen. | | | | |
